# Design and sequential jumping experimental validation of a musculoskeletal bipedal robot based on the spring-loaded inverted pendulum model

**DOI:** 10.3389/frobt.2024.1296706

**Published:** 2024-01-31

**Authors:** Yiqi Li, Yelin Jiang, Koh Hosoda

**Affiliations:** ^1^ Adaptive Robotics Laboratory, Graduate School of Engineering Science, Osaka University, Toyonaka, Japan; ^2^ Graduate School of Engineering, Kyoto University, Kyoto, Japan

**Keywords:** spring-loaded inverted pendulum model, McKibben-type pneumatic artificial muscles, musculoskeletal biped robot, robot system design, sequential jumping, ground reaction force, SLIP model simulation

## Abstract

To effectively control a robot’s motion, it is common to employ a simplified model that approximates the robot’s dynamics. Nevertheless, discrepancies between the actual mechanical properties of the robot and the simplified model can result in motion failures. To address this issue, this study introduces a pneumatic-driven bipedal musculoskeletal robot designed to closely match the mechanical characteristics of a simplified spring-loaded inverted pendulum (SLIP) model. The SLIP model is widely utilized in robotics due to its passive stability and dynamic properties resembling human walking patterns. A musculoskeletal bipedal robot was designed and manufactured to concentrate its center of mass within a compact body around the hip joint, featuring low leg inertia in accordance with SLIP model principles. Furthermore, we validated that the robot exhibits similar dynamic characteristics to the SLIP model through a sequential jumping experiment and by comparing its performance to SLIP model simulation.

## 1 Introduction

Bipedal robots capable of adapting to various environments have been a focal point of interest for many years (Hirose and Ogawa, 2007; Kaneko et al., 2011). These robots feature intricate mechanical structures and linkage mechanisms (Lohmeier et al., 2009; Kaneko et al., 2002). To control these robots, it is difficult to achieve by a model of robot whole-body dynamics, as requiring a large amount of computation and precision Scianca et al. (2020). Consequently, a common approach is to simplify the robot dynamics by approximating them with models like the inverted pendulum model or the Zero-Moment Point (ZMP) model (Vukobratović and Borovac, 2004) to address balance issues during walking and running, as exemplified by Honda’s ASIMO (Kajita et al., 2010).

Nevertheless, these control methods come with a notable drawback. They rely on trajectories generated from a simplified dynamic model, which possesses characteristics that diverge from those of the actual robot body. These disparities between the simplified model and the real-world robot properties can result in locomotion failures. Consequently, numerous researchers have endeavored to address the disparities between robot properties and these simplified models (Takenaka et al., 2009).

If the properties of bipedal robots are in alignment with the simplified model, there is no need for additional calculations to compensate for differences. various robot designs have embraced this concept by adhering to the spring-loaded inverted pendulum (SLIP) model. This model has been shown to produce ground reaction force that closely match that of humans, and has been used in the development of robot’s gaits (Blickhan, 1989; Geyer and Saranli, 2018). Some of robot design based on SLIP model are very simple robots like a pogo stick-like robot developed by Raibert et al. and Batts et al. Raibert (1986); Batts et al. (2016); Batts et al. (2017). Others have more complex structures. The ATRIAS robot designed by Oregon State University and Cassie, developed by Agility Robotics, represent successful implementations of a design approach based on the SLIP model for achieving dynamic walking and running in bipedal robots. The ATRIAS (Hubicki et al., 2016) utilizes a parallel mechanism equipped with elastic actuators. On the other hand, Cassie employs a serial mechanism characterized by a parallel linkage that incorporates elements such as leaf springs and an actuator (Abate, 2018; Apgar et al., 2018; Xiong and Ames 2018). The common design principles for these robots typically involve centralizing as much mass as possible within the body while minimizing leg inertia, based on the SLIP model.

However, each type of these robots has trade-off relations as the following points. For instance, even though robots likes ATRIAS and Cassie are designed following the simplified SLIP model, Their designs remain difficult to replicate and still required complex control strategies to control the robot (Rezazadeh and Hurst, 2020; Reher and Ames, 2021). Conversely, robots like the “Pogo stick” are relatively easy to control thanks to their simple linear joint structures. However, their ease of control comes with limitations, particularly in environments with obstacles, where their limited range of motion can pose significant challenges.

In this study, we introduce a musculoskeletal bipedal robot powered by PAMs (McKibben-type pneumatic artificial muscles) to apply the mechanistic properties of the SLIP model. In contrast to conventional robots, these robots are easy to build, there are several studies to realize pneumatic muscle-driven musculoskeletal robot (Hosoda et al., 2010; Niiyama and Kuniyoshi, 2010; Liu et al., 2018). However, previous research on pneumatic muscle-driven musculoskeletal robots did not integrate properties from simplified models into their robot design and control processes. In this paper, we take advantage of the PAM’s light weight and high output power by using a lightweight design that reduces leg inertia and concentrates 83% of the robot’s mass within a compact body.

Since jumping movements can better characterize the SLIP model. In our study, we conducted a sequential jumping experiment with the robot, employing a straightforward control approach to collect acceleration data near the robot’s center of mass and ground reaction force data. Our data analysis reveals that, with this particular design and uncomplicated control method, the robot is capable of executing jumps that closely emulate the characteristics of the SLIP model.

The remainder of this paper is structured as follows. In section II, details of the robot sequential jumping experiments are provided in Section III. Subsequently, the experimental results are presented in section IV, and based on the result finally section V provides the discussion.

## 2 Robot system design

### 2.1 Musculoskeletal structure robot design

**FIGURE 1:**
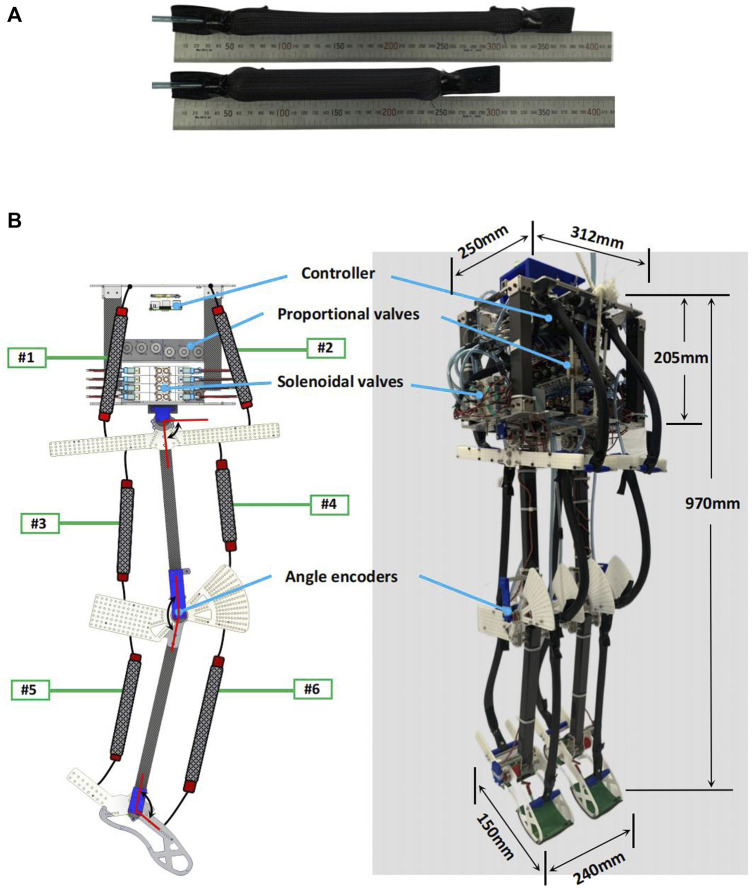
**(A)** McKibben-type pneumatic artificial muscles (PAMs). In this study PAMs are fabricated using silicone tubes, with the inner and outer diameters of the inner silicone tube measuring 14 mm and 15.6 mm, respectively. **(B)** SLIP model based musculoskeletal bipedal robot. The weight of the robot body is approximately 83% of the total weight of the robot which is similar to the SLIP model.

The musculoskeletal biped robot employs McKibben-type pneumatic artificial muscles (PAMs) which has extremely high power/weight ratios actuators (refer to Figure 1A). Each muscle is handmade and weighs merely 30 g. At the same length, PAMs made from those silicone tubes are 34% lighter than those typically made from candy rubber tubes in the previous study (Hosoda et al., 2010; Liu et al., 2018). Moreover, under the same internal air pressure conditions, PAMs made from silicone tubes demonstrate greater shrinkage.

In our investigation, we utilized a bipedal pneumatic musculoskeletal robot, as shown in Figure 1B. The robot features a compact body part, measuring 205 mm in height, 250 mm in depth, and 312 mm in width. The overall weight of the robot is 12 kg, with a height of 970 mm, depth of 150 mm, and width of 240 mm (measured as the distance between both feet), Both the thigh and the shank lengths are 340 mm. The feet measure 150 mm from heel to toe. the robot can imitate a small-size human. The robot is composed of four links: body, thigh, shank, and foot link. These links are interconnected by three hinge joints in each leg (hip, knee and ankle), constraining the motion of the robot to the sagittal plane. Each joint is actuated by a pair of monoarticular muscles acting as antagonists. Each leg is actuated by a total of six representative monoarticular muscles. The ranges of joint mobility for hip, knee and ankle are 80° ∼ 100° (flexion 10°, extension 10°), 110° ∼ 160° (flexion 50°) and 60° ∼ 105° (flexion 30°, extension 15°). Tables 1, 2 provide more detailed information regarding specific parameters, types of PAMs, and size-weight parameters related to the robot.

**TABLE 1:** Arrangement of the PAMs.

no.	PAMs length [mm]	Function	PAMs type
#1	360	hip extension	mono
#2	360	hip flexion	mono
#3	344	knee flexion	mono
#4	350	knee extension	mono
#5	380	ankle extension	mono
#6	396	ankle flexion	mono

**TABLE 2:** Robot parameters.

Parameter	Definition	Value [units]
*l*_*body*_	length of body link	235 [mm]
*l*_*thigh*_	length of thigh link	340 [mm]
*l*_*shank*_	length of shank link	345 [mm]
*l*_*foot*_	length of foot link	140 [mm]
*m*_*robot*_	weight of robot	12 [kg]
*m*_*body*_	weight of body	10 [kg]
*m*_*leg*_	weight of one leg	1 [kg]

To minimize the inertia of the robot’s legs, we chose carbon fiber tubes for both the thigh and shank segments. Additionally, in non-stress-bearing joint areas, we employed lightweight 3D printing and Polyoxymethylene boards for certain components. With the successful implementation of the lightweight design and efficient actuators, each leg of the robot now weighs merely 1.125 kg. This implies that the body of the robot carries most of the weight, accounting for approximately 83% of the total robot weight similar to the SLIP model.

### 2.2 Robot sensor and control system

The robot is equipped with a 9-axis inertial measurement unit (WitMotion Co., BWT901), pressure sensors, and magnetic rotary position sensors (ams AG Co., AS5600). These sensors capture data that is then transmitted to a Raspberry Pi 4B via custom analog-to-digital converters.

Each muscle is controlled by both a proportional valve (SMC Co., PVQ33) and a solenoid three-port valve (SMC Co., VQZ2321). Proportional valves are employed to precisely control airflow and regulate air pressure in the muscles. They receive direct control signals from a customized Pulse Width Modulation (PWM) driver board, which is operated by the Raspberry Pi 4B. Three-position solenoid valves, which have only three states - air supplying, air exhausting, and closed, are used to provide the muscle with rapid and dynamic performance. An Arduino Duo micro-controller, along with a custom signal amplifier board, is employed to control the activation of the pneumatic solenoid valves.

**FIGURE 2:**
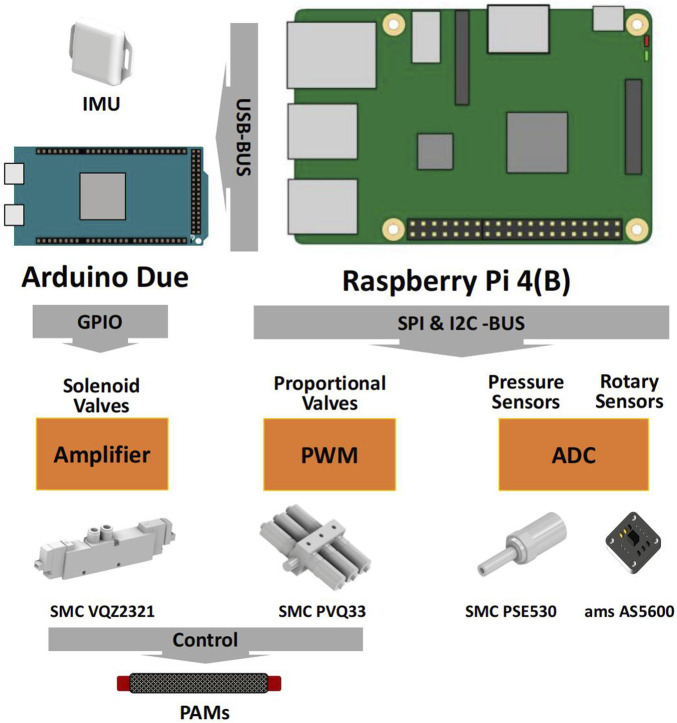
Robot sensor and control system.

The entire system reads sensor values and writes actuator commands at a rate of 200 Hz, except for the proportional valve, which is controlled at 60 Hz due to hardware constraints. The overall control system is illustrated in Figure 2.

## 3 Robot sequential jumping experiment

As the SLIP (Spring-Loaded Inverted Pendulum) model is frequently employed in analysis of jumping scenarios (Tamaddoni et al., 2010; Wensing and Orin, 2013), we devised and executed a series of sequential jumping experiments using the bipedal robot. The purpose of this experiment was to examine the characteristics and dynamic performance of the developed bipedal robot. Throughout the experiment, we collected various data from the robot, including acceleration data at the center of mass (COM) and ground reaction force (GRF) data, among others. It is important to note that we did not employ any other constraint devices to limit its movement exclusively to a sagittal plane during the experiment.

### 3.1 Data collection in experiment

During the experiment, various data from the robot were recorded, including joint angle, PAMs air pressure from joint angle sensor and pressure sensor. Euler angle and acceleration data from the Inertial Measurement Unit (IMU), all at a sampling rate of 200 Hz. ground reaction force (GRF) was measured using a force plate (Tec Gihan Co., Ltd., TF-3040) with a sampling rate of 1 kHz.

### 3.2 Robot drive pattern

**FIGURE 3:**
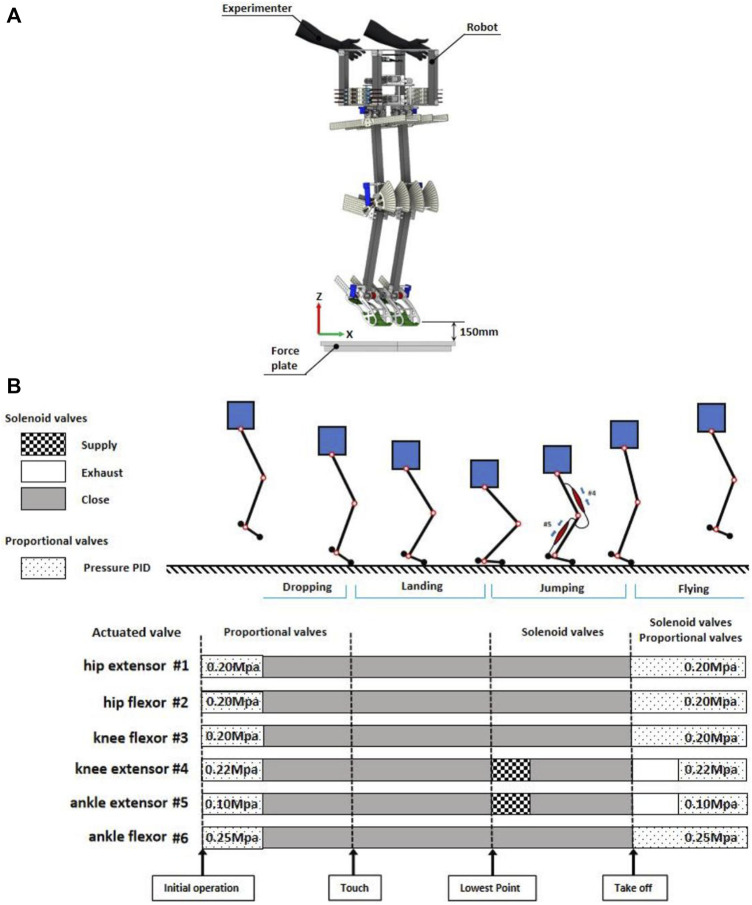
**(A)** Schematic of the initial state of the robot experiment **(B)** Robot drive pattern and states during sequential jumping.

The experimental environment and procedure are shown in Figure 3. The robot has three states in one jumping cycle: flying, landing and jumping. Robot states are determined using the data from IMU and joint angle sensors of the joints in real-time.

The muscles of robot were driven in a manner that referenced previous research (Rosendo et al., 2012; Liu et al., 2018).

#### 3.2.1 Initialization

A PID controller was used to control proportional valves to give a certain initial air pressure to each PAM. The initialization process took 1 s and the maximum air pressure error allowed during the initial phase was limited to 5%.

Muscle #1 and #2 are a pair of antagonistic muscles of the hip joint, crucial for providing high stiffness to prevent body shaking during robot jumping. These two muscles have the same specifications so the internal air pressure are set to 0.2 Mpa.

Muscles #3 and #4 constitute the antagonist muscle groups of the knee joint. In the robot’s free fall to the lowest point, the knee joint experiences the greatest force among all joints and demands high stiffness. Moreover, during the jumping motion from the lowest point, the extensor muscle of the knee joint (#4), need to contract to provide a specific force that promotes upward movement of the robot. Therefore, the internal air pressure of Muscles #3, #4 are set to 0.2 Mpa and 0.22 Mpa respectively.

The ankle joint, being the closest joint to the ground, plays a pivotal role in generating the primary jumping upward force. The ankle extension muscle (Muscle #5) is required to contract a lot, so the initial internal air pressure is very small, 0.1 Mpa. While the Muscle #6 is 0.25 Mpa.

Following the pressure initialization of PAMs, the experimenter suspended the robot mid-air at a height of 10 cm (measured from the distance between the robot’s feet and the force plate) and ensured that the robot’s body was in an upright position. Subsequently, the experimenter released the robot to commence the experiment.

#### 3.2.2 Landing

Since there are no touch sensors installed on the robot’s feet, joint angle sensors are used to determine the touch and the lowest point. We used the value of the sum of the ankle and knee joint angles to determine when the robot touches the ground and reaches the lowest point (The sum of the angles of the two joints at beginning of the change was determined to be the touching time, and at the minimum value was determined to be the lowest point time).

During this phase, all proportional and solenoid valves remain inactive. When the robot reaches its lowest point, it initiates a jump by following the control program executed by the solenoid valves. In this state, a 30 ms air supplement is provided to the two monoarticular extensor muscles (#4 and #5) in each leg to generate an upward thrust. This state concludes once the robot has completely lifted off the ground.

#### 3.2.3 Flying

As the robot commences its ascent from the ground, the air pressure in each muscle is rapidly reset within a timeframe of 130 ms. Preparing the robot for the next jumping cycle. This resetting process is accomplished through a combination of proportional and solenoid valves. Proportional valve are regulated by PID controller to manage PAMs to the specified air pressure. Additionally, solenoid valves are employed to speed up the inflating and deflating process so that air pressure of PAMs can reach the specified value faster.

Throughout all jumping phases, the muscles surrounding the hip joints contribute to a high level of stiffness, allowing the robot’s body to maintain an upright posture. This phenomenon has been extensively studied for its role in enhancing stability during various movements, including walking (Maus et al., 2010) and running (Maus et al., 2008).

## 4 Results

**FIGURE 4:**
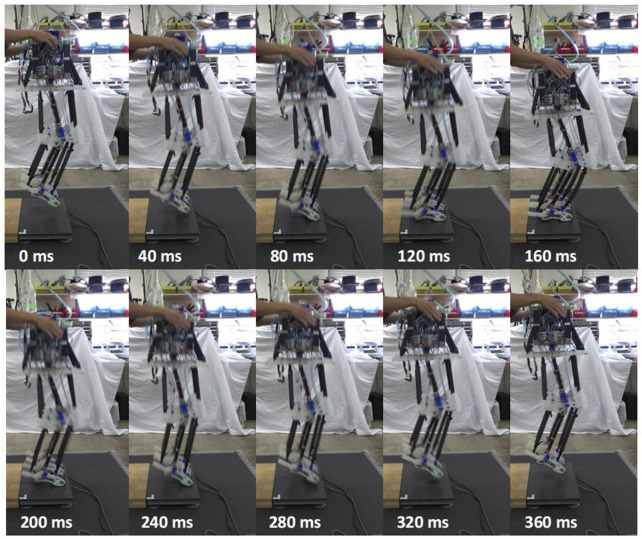
Snapshoot of robot sequential jumping. The top images show the robot free-falling from its initial state to its lowest point, and the bottom image shows it jumping into the air from its lowest point. When in contact with the ground, the robot only has its toes in contact with the ground. The heel is not in contact with the ground.

A total of ten robot jumping experiments were conducted. In each robot jumping experiment, the robot was able to complete more than four sequential jumps, and in these experiments, the robot was able to complete a maximum of 10 sequential jumps without falling. Figure 4 provides snapshots of the experiment captured at 40 ms intervals.

### 4.1 IMU data and forceplate data

**FIGURE 5:**
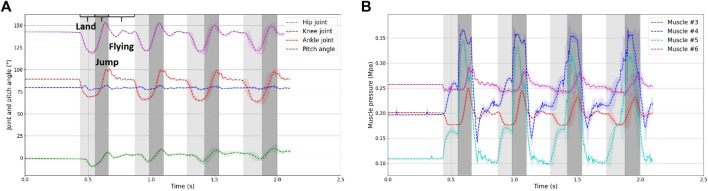
**(A)** Hip, knee and ankle joint angle during jumping experiment. Color of different part represent the landing, jumping and flying phase, respectively. **(B)** Air pressure in the antagonist muscles of the knee and ankle joints. Each muscle is able to reach a given air pressure in a very short period during flying phase under the action of the PID of the proportional valve.

To derive the average data, we collected information from ten jumping experiments and concentrated our analysis on the initial four jumping cycles in each jumping experiment. Figure 5 reveals that the robot’s rotational angle in the pitch direction is confined to just ±10°. This observation suggests that the robot’s body maintains a predominantly upright posture throughout the jumping process.

**FIGURE 6:**
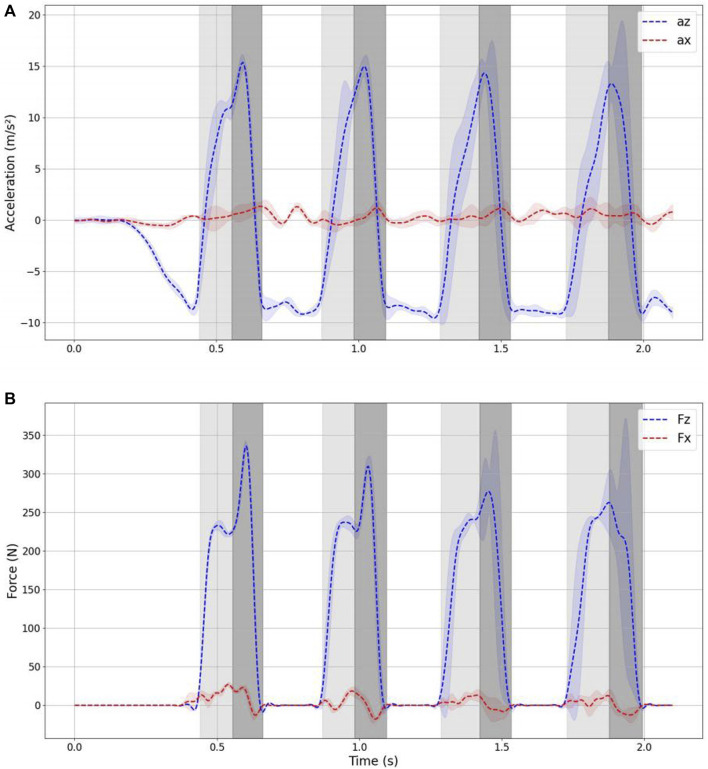
Acceleration and ground reaction force during jumping. **(A)** Accelerations in x-z axis measured from IMU **(B)** ground reaction force in x-z axis measured from forceplate.

Figure 6 displays the acceleration and force data recorded by the IMU and force plate. To mitigate the influence of noise, we applied a low-pass filtering procedure to the data with a cutoff frequency of 20 Hz.

Analyzing the ground reaction acceleration data, it is evident that the robot consistently follows a vertical jumping motion. The yellow segment represents the landing phase, while the green segment corresponds to the jumping phase. As depicted in the figure, the standard deviation across the ten experiments remains relatively low for the initial three jumping cycles but noticeably increases during the fourth jumping cycle. This variation can be attributed to differences in the initial conditions of the experiments, such as inaccuracies in the initial muscle air pressure and variations in the initial drop height. These discrepancies cumulatively lead to disparities in the timing of landing and takeoff during subsequent jumps.

Based on the data, we can observe the following time duration:

• The time from the moment of touching the ground to reaching the lowest point is approximately 120,130 ms.
• The time from the lowest point to completely leaving the ground is approximately 100,110 ms.
• The total time spent in the air during a single jump cycle is approximately 200 ms.

As evident from the data, the force does not reach its maximum at the lowest point but instead achieves its peak roughly 30 ms after hitting the lowest point. This phenomenon occurs because the solenoid valve is activated for a duration of 30 ms after the robot reaches its lowest point within the jumping cycle. The delayed activation of the solenoid valve is responsible for the force reaching its maximum at that specific time after the lowest point has been reached.

### 4.2 Comparison of the measured and calculated accelerations

In order to verify whether the robot possesses one of the crucial propertiy of the SLIP model, which enables the center of mass (COM) dynamics to represent the entire robot’s dynamics, we make the assumption that while in the jumping phase, due to the small inertia of the robot’s legs, the reaction force of the ground acts entirely on the center of mass of the robot. With this assumption, we proceed with the calculation of acceleration based on this premise. We then compare the calculated COM acceleration from force data with the actual acceleration measured at the COM. This comparison helps assess the degree to which the robot’s behavior aligns with the SLIP model’s characteristics.

The force data recorded by the force plate is processed using Eqs 1, 2 to compute the acceleration when the robot is in contact with the ground. When the robot is in mid-air and not in contact with the ground, the acceleration is set to −9.8 *m*/*s* ^2^. In these equations:*m*_*r*_ represents the mass of the robot. *g* is the acceleration due to gravity. *acc*, *xcalculated* and *acc*, *zcalculated* are the calculated accelerations in the x-axis and z-axis directions, derived from the force data.

$${acc\;x}_{\mathrm{calculated}}=\frac{F_{x}}{m_{\mathrm{robot}}}$$ (1)

$${acc\;z}_{\mathrm{calculated}}=\frac{F_{z}}{m_{\mathrm{robot}}}-g$$ (2)

**FIGURE 7:**
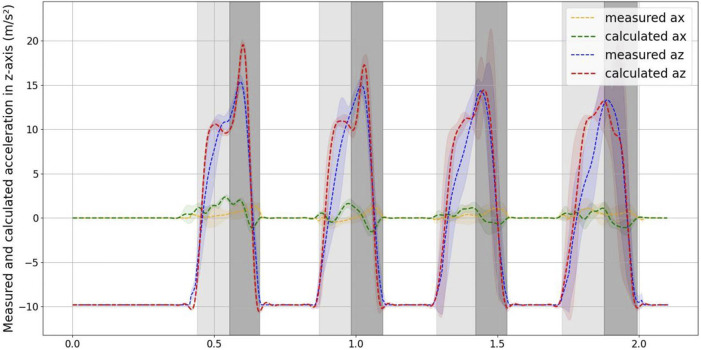
Measured and calculated accelerations in x-z axis. The distribution of the x-axis measured and calculated acceleration the values are 0.195 ± 0.358 *m*/*s* ^2^ and 0.188 ± 0.636 *m*/*s* ^2^. The distribution of the z-axis measured and calculated acceleration the values are −2.909 ± 8.850 *m*/*s* ^2^ and -2.594 ± 9.566 *m*/*s* ^2^.

**FIGURE 8:**
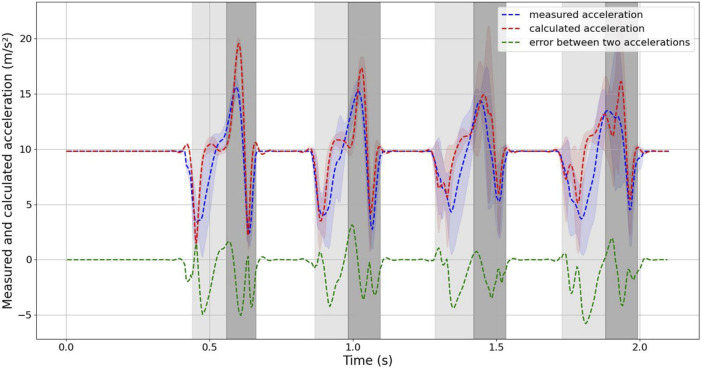
Comparison of measured and calculated accelerations during robot jumping. The integrated acceleration value and the error.

Figure 7 displays a comparison between the measured and calculated accelerations in the *x* and *z*-axes, with positive and negative values indicating the direction of acceleration. Additionally, Figure 8 depicts the value comparison of the combined acceleration, $\left|accmeasured\right|$ and $\left|acccalculated\right|$ , obtained from Eqs 3, 4. The distribution of measured and calculated integrated acceleration values, it is evident that the mean values are 9.222 ± 2.499 and 9.938 ± 2.294 (mean ± s.d.) *m*/*s* ^2^.

As evident from the figure, the curve of the measured acceleration exhibits inflection points when the robot just makes contact with the ground, jumps, and leaves the ground. In contrast, the calculated acceleration curve does not display this phenomenon. This difference can be attributed to the fact that the acceleration is measured at 200 Hz, whereas the ground reaction force is measured at 1,000 Hz, resulting in a smoother acceleration curve calculated from the ground reaction force.

The error is largest when the robot just makes contact with the ground and when it jumps from the lowest point. This is attributed to the fact that the instantaneous acceleration is highest in these instances, and the distinct rates of data collection contribute to significant differences between the measured and calculated values.

$$\left|{acc}_{\mathrm{measured}}\right|=\sqrt{{\left({acc\;x}_{\mathrm{measured}}\right)}^{2}+{\left({acc\;z}_{\mathrm{measured}}\right)}^{2}}$$ (3)

$$\left|{acc}_{\mathrm{calculated}}\right|=\sqrt{{\left({acc\;x}_{\mathrm{calculated}}\right)}^{2}+{\left({acc\;z}_{\mathrm{calculated}}\right)}^{2}}$$ (4)

### 4.3 The vectors of COM and GRF direction during one jumping cycle

**FIGURE 9:**
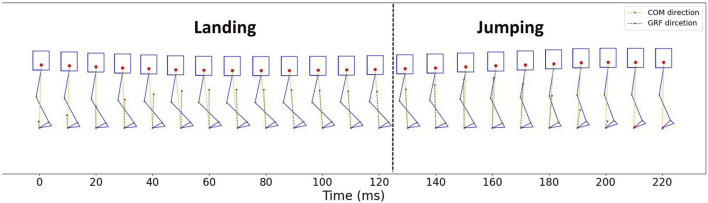
COM and GRF vectors direction in one jumping cycle.

Figure 9 illustrates the robot’s pose, the vectors of ground reaction force (GRF) computed using Eq. 5, and the vectors of the Center of Mass (COM) every 10 ms throughout a single jumping cycle in a series of jumps. This kinematic calculation is derived from joint angle data, allowing the determination of the robot’s orientation when it is in contact with the ground.

The red dotted line in the figure represents the point of contact with the ground in the direction of the Center of Mass (COM), pointing from the toe of the foot to the center of gravity. The green dotted line represents the direction of the ground reaction force (GRF). The average angle between the direction vector of COM and GRF, as described in Eq. 6, is 3.565 ± 3.706 (mean ± standard deviation) degrees (°).

$$\vec{GRF}=\vec{F_{x}}+\vec{F_{z}}$$ (5)

$$angle=∠\left(\vec{COM},\vec{GRF}\right)$$ (6)

**FIGURE 10:**
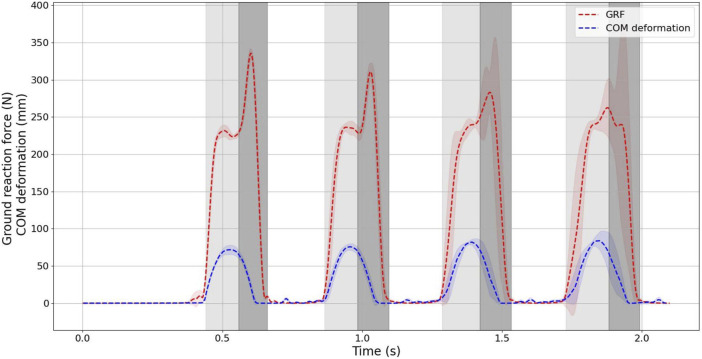
Ground reaction force and COM deformation.

Figure 10 expresses the relationship between ground reaction force (GRF) and center of mass (COM) deformation during the sequential jumping. To facilitate a visual comparison between the jumping motion of the designed robot and the ideal SLIP (Spring-Loaded Inverted Pendulum) model, we considered the robot’s leg as the spring-loaded leg in the SLIP model and calculated the stiffness using Eq. 7. The average value of the calculated stiffness for ten sequential jumps, denoted as *k*_*spring*_, is 3.413 ± 1.151 *N*/*mm*.

$$k_{\mathrm{spring}}=\frac{d\left|\vec{GRF}\right|}{d\left|\vec{COM}\right|}$$ (7)

To assess how closely the jumping behavior of the designed robot aligns with that of the SLIP (Spring-Loaded Inverted Pendulum) model, a simulated SLIP dynamical model for hopping was created using the parameters from Table 3. These parameters were derived and computed based on the characteristics of the designed robot. The simulation employed the ode45 solver within MATLAB/SIMULINK 2020a, with a time step set to match the robot’s data sampling interval of 0.005 s. This simulation allows for a comparative analysis of the robot’s jumping performance in relation to the idealized SLIP model.

**TABLE 3:** SLIP model parameters in simulation.

SLIP mdoel parameter	symbol	Value [units]
trunk mass	m	12 [kg]
leg rest length	*l*_0_	800 [mm]
leg stiffness	k	3.4 [N/mm]
initial height	*h*_0_	100 [mm]
time step	*t*_*step*_	0.005 [s]

**FIGURE 11:**
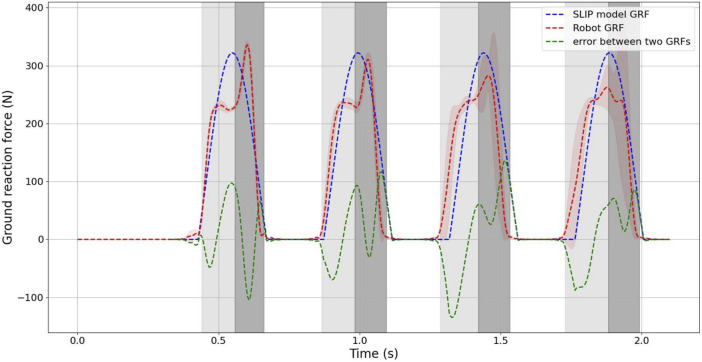
Comparison of the SLIP model and designed robot sequential jumping. The Value of ground reaction force during sequential jumping and the error.

Figure 11 provides a comparison of the ground reaction force (GRF) during jumping between the SLIP model simulation and the designed robot. The results clearly demonstrate that in the SLIP model dynamical simulation, the ground contact time and air time are 0.244 s and 0.202 s, respectively. For the robot, the ground contact time varies from 0.22 s to 0.24 s, while the air time ranges from 0.19 s to 0.21 s. It is evident that the timing of the real robot closely aligns with the touchdown and takeoff phases of the SLIP model simulation.

It is important to highlight that in the SLIP model simulation, the ground reaction force (GRF) reaches its maximum at the lowest point, whereas for the robot, there is a delay of approximately 30 ms before the GRF reaches its maximum due to the solenoid valve. However, the maximum value of the GRF is essentially the same for both the robot and the SLIP model. As evident from the error curves in the figure, the primary cause of error in the first two jump cycles is the delay in reaching the maximum value of the ground reaction force.

Although the GRF distributions for both the SLIP model and the robot are remarkably similar, the maximum GRF value for the robot is slightly smaller than that of the SLIP model during jumping. This discrepancy is primarily attributed to increased errors in the robot’s later two jumping cycles, resulting in a slightly lower mean GRF value for the robot compared to the SLIP model simulation. The error curves also reveal that the main discrepancy between the model and the experiment stems from the misalignment of jump cycle times.

**FIGURE 12:**
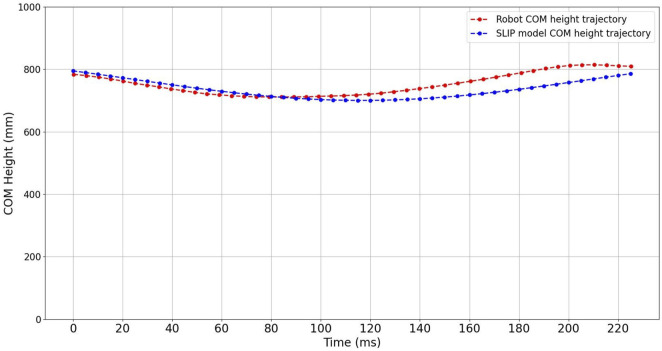
COM height trajectory comparison of the SLIP model and designed robot during one jumping cycle.

To acquire a more comprehensive understanding of the distinctions between the jumps of the robot and the SLIP model simulation, it is essential to have not only compared the GRF but have also examined the trajectory of the center of gravity at the moment of ground contact. This additional analysis allows for a more comprehensive visualization of the differences and similarities in the motion between the two systems during the jumping process. Given that it is a vertical jump, the comparison of the trajectory at the height of the center of gravity is a relevant and meaningful approach. As demonstrated in Figure 12, the trajectories of the center of gravity height exhibit a high degree of similarity between the robot and the SLIP model. This result demonstrates further supports the conclusion that the robot’s jumping behavior closely resembles that of the SLIP model. During the phase from touching the ground to reaching the lowest point, the height trajectories of the center of gravity for both the robot and the SLIP model are largely identical. However, in the phase from the lowest point to takeoff, differences become pronounced. The robot utilizes valve control to execute its jump, whereas the ideal SLIP model relies on the passive behavior of spring-loaded legs. This distinction in jumping mechanisms leads to a more noticeable difference between the two trajectories during this phase.

Based on the analysis and comparisons provided, it can be concluded that the dynamic performance of the designed robot closely approximates that of the SLIP model. This suggests that the robot’s jumping behavior corresponds well with the fundamental principles and dynamics of the SLIP model.

## 5 Discussion

In this paper, we have designed a musculoskeletal robot utilizing PAMs as actuators. The utilization of lightweight PAMs and the implementation of a lightweight leg design have significantly reduced the inertia of the robot’s legs. The mass distribution of the robot, designed in accordance with the SLIP model, is highly concentrated, with approximately 83% of the mass centered within the robot’s compact body. To validate the dynamic performance of the robot and assess the differences compared to the dynamic performance of the SLIP model, we conducted robot jumping experiments using a straightforward control approach. These experiments were conducted to collect acceleration data near the robot’s center of mass and to record ground reaction force data.

The evaluation of the dynamic performance of the designed robot was based on the two fundamental characteristics of the SLIP model, as outlined in references (Blickhan, 1989; McMahon and Cheng, 1990). One crucial property of the SLIP model is that it represents the body as a point mass situated at the Center of Mass (COM), while the legs are conceptualized as massless springs. This characteristic implies that the dynamics of the point mass are entirely generated by the reaction forces exerted by the legs as they come into contact with the ground.

During the experiment involving vertical jumping, the measured and calculated accelerations in the z-axis direction are generally similar. However, the data in the x-axis direction is more susceptible to noise, resulting in a relatively larger deviation between the measured and calculated accelerations. To reduce the effect of noise and variations in the x-axis direction, you have compared the combined measured and calculated accelerations, as depicted in Figure 8. This comparison has revealed a very high degree of similarity between the two, which helps provide a more accurate assessment of the robot’s performance. The agreement between the calculated COM acceleration, derived from the ground reaction force data and the robot’s mass, and the measured COM acceleration is notably high. Therefore, we concentrated as much mass as possible in the center of mass and reduced the mass of the legs in a design that gives the robot one property of the SLIP model. In the future, we can consider only the dynamics of COM when designing the control strategy for this robot, which will simplify the design of the controller.

Another important characteristic of the SLIP model is that the reaction force generated by the massless spring leg with the ground is consistently directed toward the COM. In our experiments, we found a simple muscles drive patterns by try and error, without involving intricate model-based control of individual muscles. However, it is evident from Figure 9, the direction of the GRF is predominantly directed towards the COM during jumping. This result is consistent with the property of the approximated SLIP model that were obtained, suggesting that the robot legs have been approximated as a spring like that in the SLIP model. And as shown from the comparison of the robot with the SLIP model simulation in Figure 11, 12, it is evident that the robot can exhibit comparable dynamic performance to that of the SLIP model using a simple straightforward control approach.

In contrast to previously engineered robots inspired by the SLIP model, which featured fixed leg stiffness and faced challenges in adjusting it Owaki et al. (2010), Hereid et al. (2014), our robot possesses the ability to modify leg stiffness by altering the air pressure in the Pneumatic Artificial Muscles (PAMs). In this way, the designed robot can exhibit different dynamic behaviors without the need to change the mechanics. Although prior musculoskeletal robots powered by pneumatic muscles were characterized by a lighter overall weight and a greater number of muscles, none of them adhered to the design principles of the SLIP model. Those robots were unable to jump more than twice in sequential jumping (Hosoda et al., 2010; Niiyama and Kuniyoshi, 2010; Liu et al., 2018). Our designed robot was able to jump more than four times sequential jumping, and the highest jump times was up to 10 times. Our research bridges the gap between musculoskeletal robots and SLIP models.

Many researchers have investigated the control of jumping, walking and running based on the SLIP model. One of the easiest ways to control the jump height and distance of a SLIP model is to adjust the stiffness and angle of the legs (Raibert et al., 1983). Altering these parameters has the potential to influence the motion’s dynamic characteristics, as highlighted in studies such as (Geyer and Saranli, 2018).

In this study we only used a simple straightforward control without any feedback control. In the future, we can enhance control by developing models for the Pneumatic Artificial Muscles (PAMs) and integrating those models with a SLIP model. By controlling and thus changing the stiffness of the legs, we can enable the robot to exhibit different dynamic properties such as walking and running motion guided by the control strategy derived from the SLIP model.

## Data Availability

The raw data supporting the conclusion of this article will be made available by the authors, without undue reservation.
